# Moderately increased albuminuria, chronic kidney disease and incident dementia: the HUNT study

**DOI:** 10.1186/s12882-019-1425-8

**Published:** 2019-07-12

**Authors:** Jessica Mira Gabin, Solfrid Romundstad, Ingvild Saltvedt, Jostein Holmen

**Affiliations:** 1 HUNT Research Centre, Department of Public Health and Nursing, Norwegian University of Science and Technology (NTNU), Trondheim, Norway; 2 Department of Internal Medicine/Nephrology, Levanger Hospital, Health Trust Nord-Trøndelag, Levanger, Norway; 3 Department of Clinical and Molecular Medicine, Norwegian University of Science and Technology, Trondheim, Norway; 4 Department of Neuromedicine and Movement science, Norwegian University of Science and Technology, Trondheim, Norway; 5 Department of Geriatrics, St Olavs Hospital, University Hospital of Trondheim, Trondheim, Norway

**Keywords:** Epidemiology, Chronic kidney disease, Moderately increased albuminuria, Dementia, Alzheimer disease, Vascular dementia

## Abstract

**Background:**

Epidemiologic studies has shown an association of albuminuria and low estimated glomerular filtration rate (eGFR) with dementia, but the findings are inconsistent. This study examines the association between eGFR, MA with dementia and its subtypes: AD, VaD, a mixture of AD/VaD, and other dementias.

**Methods:**

Data from the second wave of the HUNT 2 Study (1995–1997) were linked with a dementia register known as the Health and Memory Study (HMS) collected during 1995–2011 in Nord-Trøndelag County, Norway. Dementia was ascertained using World Health Organization’s ICD-10 criteria into subtypes: AD,VaD, mixed AD/VaD, and other dementia. eGFR and its association with dementia was examined in 48,508 participants of the HUNT Study, of which 668 were diagnosed with all-cause dementia. Association between MA and dementia were studied in a subset of 7024 participants, and 214 were diagnosed with all-cause dementia. Cox regression models were conducted analyzing the association between dementia and MA using albumin creatine ratio (ACR). Cox regression models and Fine-Gray models were used to examine the association between dementia and eGFR.

**Results:**

A positive association was found between increasing ACR and dementia. ACR in the fourth quartile (> 1.78 mg/mmol) with increased hazard ratio of VaD, 3.97 (1.12 to 14.07), compared with ACR in the first quartile (<.53 mg/mmol). There was no association between eGFR and dementia or its subgroups.

**Conclusions:**

Our results strengthens the hypothesis that vascular mechanisms may affect both kidney and brain as an association between MA and dementia was found. However, eGFR was not significantly associated with dementia independent of diabetes mellitus or hypertension.

**Electronic supplementary material:**

The online version of this article (10.1186/s12882-019-1425-8) contains supplementary material, which is available to authorized users.

## Background

As dementia remains to be irreversible, studies continue to examine modifiable risk factors aimed at prevention [1]. The ageing population is growing, and the number of patients with chronic kidney disease (CKD) is expected to increase [2]. Estimated glomerular filtration rate (eGFR) and albuminuria are established markers in identifying CKD or kidney damage [2, 3]. CKD is defined as a reduced GFR, increased urinary albumin excretion, or both, and is an increasing public health issue [4]. Studies have found CKD to be associated with an increase in cardiovascular disease risk and worse cognitive performance [5, 6]. Both CKD and dementia share similar risk factor profiles consisting of hypertension, diabetes mellitus, stroke, myocardial infarction, and hyperlipidemia [1, 7]. However, the association between CKD and dementia remains unclear [8]. The kidneys and the brain are susceptible to vascular damage as they are exposed to high-volume blood flow. Epidemiological studies have found albuminuria and low GFR associated with Alzheimer disease (AD) and Vascular dementia (VaD) [9, 10]. However, findings have been mixed, where adverse and no associations have been published [11–14].

An early risk marker of renal endothelial dysfunction is known as moderately increased albuminuria [formerly called microalbuminuria (MA)]. MA is a term to describe moderate amounts of albumin present in the urine, and studies have shown it to be an early risk marker of cardiovascular disease [15]. The importance of MA in cardiovascular disease has been validated, and although it is uncertain, a vascular mechanism is presumably shared by both the kidney and brain and has been suggested as the cause of any shown association [16].

The aim of this study was to evaluate the association between MA using albumin creatinine ratio (ACR) at baseline and the risk for incident AD, VaD and a mixture of these in a prospective study in a population-based cohort. In addition, we examined eGFR, to see if associations differed across samples in the varying stages of CKD.

## Methods

### Study population

**Fig. 1:**
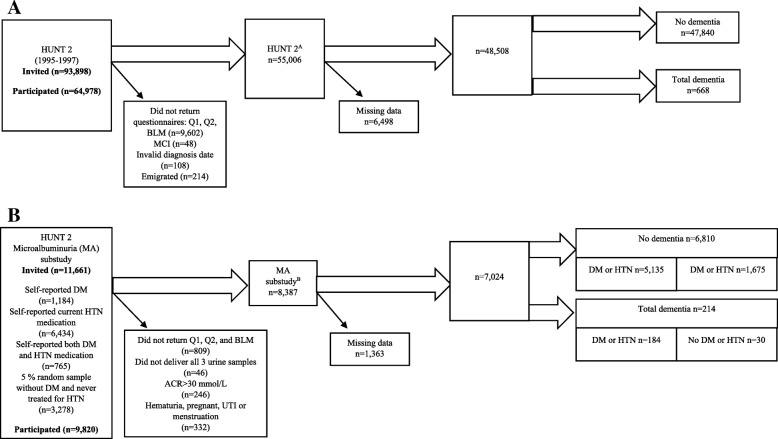
Flow chart indicating the number of invited participants and selection criteria from the (**a**) HUNT 2 study, and (**b**) the MA substudy. Abbreviations: *Q1* HUNT 2 self-report questionnaire one, *Q2* HUNT 2 self-report questionnaire two, *BLM* HUNT 2 data on blood samples and standardized health screenings, *MCI* mild cognitive impairment, *MA* moderately increased albuminuria, *DM* diabetes mellitus, *HTN* hypertension, *ACR* albumin creatinine ratio, *UTI* urinary tract infection

The general health survey known as HUNT 2 (1995–1997) invited all residents ≥20 years (*N* = 93,898) residing in Nord-Trøndelag County, Norway (see Fig. 1a). Data were collected using questionnaires, clinical measurements and collection of blood and urine samples, and 64,978 (69.2%) residents participated. The details of the HUNT study design have been published previously [17]. Participants who emigrated out of the County (*n* = 214), lacked a dementia diagnosis date (*n* = 108), diagnosed with prevalent mild cognitive impairment (MCI) (*n* = 48), or those who did not provide both clinical examination and self-reported questions on hypertension, diabetes mellitus (DM), stroke, smoking habits, alcohol consumption, and physical activity were removed from the sample (*n* = 9602). A total of 55,006 participated in both questionnaires and clinical examinations, however, a further 6498 participants were removed due to missing covariate data, resulting in 48,508 participants included in the present study examining the association between CKD and dementia. 668 participants were diagnosed with dementia and 47,840 were not diagnosed with dementia.

### MA substudy

Participants of HUNT 2 who self-reported DM and/or treated hypertension (HTN), and a randomly selected, non-diabetic/non-treated hypertensive sample were included in MA screening, and asked to deliver three urine samples from three consecutive days. Details of the MA screening have been published [15]. The flow chart in Fig. 1b shows identical selection and inclusion criteria for both CKD and MA, where participants lacking either questionnaires, clinical examination, or relevant data on dementia diagnosis were excluded (*n* = 809). A further 46 did not deliver all three urine samples, 246 had an ACR over 30 mmol/L, and 332 were removed from the sample, as participants confirmed self-reported history of hematuria, menstruation, or pregnancy. 8387 remained in the sample and 1363 had missing covariate data and were removed. 7024 participants had complete data and encompass this study sample. Subgroups were created to examine those diagnosed with dementia and DM or treated for HTN (*n* = 184) and those diagnosed with dementia and without DM or treatment for HTN (*n* = 30). Similarly, 5135 participants without dementia and with DM or treatment for HTN, and 1675 controls were without DM and were not treated for HTN.

### Clinical examination and laboratory procedures

In HUNT 2, the clinical examination was conducted in survey stations. Height and weight was used to generate body mass index (BMI) based on participants wearing light clothes without shoes: height to the nearest centimeter and weight to the nearest half kilogram. Pulse, systolic (SBP) and diastolic blood pressure (DBP) were measured three times using a Dinamap 845XT (Critikon) based on oscillometry. Non-fasting blood sampling occurred at survey stations during HUNT 2. Fresh serum samples were analyzed using Hitachi 91 Autoanalyser (Hitachi, Mito, Japan) and described in detail previously [18]. Serum concentration of creatinine was used to calculate eGFR using the Chronic Kidney Disease Epidemiology Collaboration equation (CKD-EPI). Further description of examination and laboratory procedures have been described in detail according to standardized protocols [17].

### Dementia ascertainment

A parallel study, known as the health and memory study (HMS) of Nord Trøndelag County, took place from 1995 to 2011. The HMS study is a registry of persons diagnosed with dementia in the county from hospital memory clinics between 1995 and 2010; or from nursing home ascertainments performed in 2010–2011. Dementia ascertainment was uniform amongst hospital and nursing home panels. Ascertainment was based on clinical examination, patient, caregiver history, and diagnostic imaging. The ICD-10 classification system was thereafter applied for diagnoses of AD, VaD, a mixture of AD and VaD. Other dementias refers to patients with frontotemporal dementia (FTD), dementia of Lewy bodies (LBD), and unspecified. Establishing the date of the diagnosis of dementia was determined by a panel of specialists in geriatric medicine and psychiatry in the two hospitals or by asking nursing home staff or next of kin the number of months residents of nursing homes had displayed symptoms of memory loss. Details of this cohort have been previously published [19].

### Urine sampling

Participants of the MA screening received a unit with three plastic receptacles for three first morning urine samples, three transport tubes, and pre-paid return envelopes addressed to the laboratory. Written instructions provided information how to collect the sample. Participants responded to specific questions pertaining to history of a urinary tract infection during the last week, persistent hematuria over the last year, and whether women were pregnant or menstruating at collection time. Fresh urine samples were analyzed using Hitachi 91 Autoanalyser (Hitachi, Mito, Japan). Urine albumin and creatinine were measured by an immunoturbimetric method (antihuman serum albumin from Dako Norway, Oslo) and Jaffe method [15].

### Follow-up and endpoints

The national identification number of every Norwegian inhabitant enables linkage between the Death Registry at Statistics Norway to determine vital status (alive, emigrated, and dead). Each participant contributed in person-years from the date of study entry until the date of death, date diagnosed with dementia or controls who survived to the end of the study (April 23rd, 2011).

### Data analysis

First, we analyzed the association between eGFR and dementia and its subtypes AD, VaD, mixed AD/VaD, and other dementia. The Cox proportional hazards model was used to calculate age-and multivariate-adjusted hazard ratios (HRs) with 95% confidence interval (CI) for dementia according to declining eGFR, examined both continuously and using the following categorical cutoffs with eGFR: > 90, 60–89.9, 30–59.9, and < 30. Repeated analyses using the Cox proportional hazards model were performed for ACR. ACR was calculated using the mean of three ACRs and was log transformed when used as a continuous variable in analyses. ACR was also examined categorically as quartiles: 0–.53, .54–.87, .88–1.77, and ≥ 1.78 mg/mmol. Participants’ age was used as a continuous variable in analyses. Level of education was categorized according to primary (seven years or less), secondary (seven to nine years), and upper secondary education (> 10 years). SBP and DBP were calculated as the mean of the second, third of three measurements, and used as continuous variables in analyses. Non-fasting glucose, cholesterol, and iron were scored as continuous variables. Self- report data on history of myocardial infarction (MI), angina, stroke, DM, and smoking were dichotomous variables, and subjective health status was used in analyses categorically according to poor, not so good, good, and very good. The independent-samples t-test and Pearson’s chi squared were used to compare the means between groups for continuous and categorical variables. Four sets of hazard regression models were performed for each endpoint in a hierarchy. Effect modification was examined by testing the age and sex multiplied with both eGFR and log transformed ACR in multi-adjusted models. Finally, supplementary analyses were performed to examine the assumption that lower levels of eGFR comes an increased risk of dying, and death as a competing risk. Competing risk regression was examined with Fine-Gray models using proportional subdistribution hazards (SHs) with 95% CI in models. SPSS Version 25 Software performed analyses shown in Tables 2 and 4. STATA version 14 performed supplementary analyses shown in supplementary material 3.

## Results

### eGFR

During the study period, 7606 died and 668 developed dementia. The mean age of the study sample was 49.5 ± 16.7 years, and mean eGFR was 78.8 ± 16.2 ml/min/1.73 m^2^. Persons diagnosed with dementia were older, with reduced eGFR, higher SBP and DBP, and higher prevalence of self-reported cardiovascular disease. Additional characteristics of this sample are shown in Table 1. There was no statistically significant association between eGFR and dementia or its subgroups when examined in hazard regression models, see Table 2. There were interactions between age and eGFR in dementia and its subgroups. Splitting the sample at median age < 72.1; > 72.2, did not change the results in either samples. An age interaction was observed in VaD, however did not provide supportive evidence of any association when examining women and men separately (data not shown). Supplementary analyses with competing risk regression were performed to assess the effect of death on the association between eGFR and dementia, and are shown in Additional file 3. The proportional assumption for the Fine-Gray model was met by testing for time-by-status interaction in the multivariate analysis.

**Table 1:** Characteristics of the study sample examining estimated glomerular filtration rate (eGFR)

HUNT 2 (1995–1997)	No dementia	Total Dementia	Combined AD/VaD/Mixed AD/VaD	AD	VaD	Mixed AD/VaD	Other Dementia	*P value* ^*A*^
Total study population, n	47,840	668	528	348	112	68	140	
eGFR, mean (SD)	78.99 (16.15)	63.89 (12.39)	63.13 (12.40)	62.79 (12.12)	64.97 (14.39)	61.88 (9.84)	66.74 (11.91)	*.00*
4 stages eGFR, n (%)								*.00*
> 90	11,708 (24.5)	16 (2.4)	12 (2.3)	8 (2.3)	4 (3.6)	0	4 (2.9)	
60.0–89.9	30,347 (63.4)	397 (59.4)	306 (58.0)	196 (56.3)	68 (60.7)	42 (61.8)	91 (65.0)	
30.0–59.9	5697 (11.9)	251 (37.6)	207 (39.2)	143 (41.1)	38 (33.9)	26 (38.2)	44 (31.4)	
< 30	88 (.2)	4 (.6)	3 (.6)	1 (.3)	2 (1.8)	0	1 (.7)	
Diabetes Mellitus, n (%)	1307 (2.7)	34 (5.1)	31 (5.9)	18 (5.2)	8 (7.1)	5 (7.4)	3 (2.1)	*.00*
Anti-hypertensive tablets, ever, n (%)	6476 (13.5)	211 (31.6)	173 (32.8)	102 (29.3)	48 (42.9)	23 (33.8)	38 (27.1)	*.00*
Sex, Female, n (%)	25,806 (53.9)	410 (61.4)	341 (64.6)	238 (68.4)	58 (51.8)	45 (66.2)	69 (49.3)	*.00*
Age at HUNT 2 (1995–1997), mean (SD)	49.24 (16.55)	70.58 (7.54)	71.26 (6.99)	71.62 (6.80)	69.82 (8.19)	71.84 (5.38)	68.02 (8.93)	*.00*
Time to debut, years, mean (SD)		7.15 (3.63)	7.15 (3.65)	7.20 (3.76)	7.41 (3.53)	6.45 (3.16)	7.16 (3.57)	
Education, n (%)								*.00*
Primary	17,234 (36.0)	458 (68.6)	367 (69.5)	240 (69.0)	77 (68.8)	50 (73.5)	91 (65.0)	
Completed secondary	26,830 (56.1)	195 (29.2)	151 (28.6)	104 (29.9)	30 (26.8)	17 (25.0)	44 (31.4)	
Completed upper secondary	3776 (7.9)	15 (2.2)	10 (1.9)	4 (1.1)	5 (4.5)	1 (1.5)	5 (3.6)	
Cholesterol (mmol/L), mean (SD)	5.87 (1.25)	6.65 (1.24)	6.70 (1.24)	6.72 (1.23)	6.65 (1.5)	6.64 (1.29)	6.46 (1.21)	*.00*
Non-fasting blood glucose (mmol/L), mean (SD)	5.42 (1.46)	5.88 (1.78)	5.91 (1.79)	5.89 (1.85)	6.03 (1.84)	5.77 (1.37)	5.78 (1.74)	*.00*
Serum iron (), mean (SD)	16.51 (6.31)	16.08 (5.74)	15.99 (5.79)	16.07 (5.89)	15.55 (5.71)	16.28 (5.55)	16.45 (5.58)	*.06*
Body Mass Index (kg/m^2^) mean (SD)	26.32 (4.07)	27.03 (4.11)	26.96 (4.04)	26.76 (4.03)	27.62 (4.39)	26.91 (3.34)	27.28 (4.36)	*.00*
Pulse (beats/min), mean (SD)	72.96 (12.56)	73.63 (13.87)	73.70 (13.71)	73.77 (14.44)	72.41 (11.77)	75.50 (12.72)	73.36 (14.53)	*.21*
Systolic BP (mmHg), mean (SD)	136.86 (21.23)	153.27 (23.23)	153.94 (23.52)	153.96 (23.63)	153.63 (22.17)	154.34 (25.34)	150.72 (21.99)	*.00*
Diastolic BP (mmHg), mean (SD)	80.02 (12.01)	84.68 (12.56)	84.73 (12.56)	83.97 (12.50)	86.47 (12.27)	85.69 (13.13)	84.50 (12.62)	*.00*
Myocardial Infarction, n (%)	1444 (3.0)	40 (6.0)	32 (6.1)	18 (5.2)	10 (8.9)	4 (5.9)	8 (5.7)	*.00*
Angina Pectoris, n (%)	2178 (4.6)	90 (13.5)	71 (13.4)	46 (13.2)	15 (13.4)	10 (14.7)	19 (13.6)	*.00*
Stroke, n (%)	809 (1.7)	25 (3.7)	21 (4.0)	7 (2.0)	12 (10.7)	2 (2.9)	4 (2.9)	*.00*
Daily Smoker, n (%)	13,425 (28.1)	110 (16.5)	80 (15.2)	44 (12.6)	25 (22.3)	11 (16.2)	30 (21.4)	*.00*
Subjective health status								*.00*
Poor, n (%)	752 (1.6)	13 (1.9)	12 (2.3)	7 (2.0)	5 (4.5)	0	1 (.7)	
Not so good, n (%)	11,646 (24.3)	303 (45.4)	239 (45.3)	149 (42.8)	55 (49.1)	35 (51.5)	64 (45.7)	
Good, n (%)	27,659 (57.8)	323 (48.4)	260 (49.2)	181 (52.0)	47 (42.0)	32 (47.1)	63 (45.0)	
Very good, n (%)	7783 (16.3)	29 (4.3)	17 (3.2)	11 (3.2)	5 (4.5)	1 (1.5)	12 (8.6)	

^A^*P*-values are derived from *t* tests for continuous variables and *x*^2^ tests for the binary variables comparing means between total dementia and no dementia

**Table 2:** Cox proportional regression models on the association of glomerular filtration rate (eGFR) and dementia. Hazard ratios are presented with 95% confidence intervals

		Total Dementia	NC^A^	Combined AD, VaD, Mixed AD/VaD	NC	AD	NC	VaD	NC	Mixed AD/VaD	NC	Other Dementia	NC
	eGFR		668		528		348		112		68		140
Model 1^c^	> 90	Ref		Ref		Ref		Ref		Ref		Ref	
	69.0–89.9	9.96 (6.04–16.42)		10.52 (5.76–18.26)		9.88 (4.87–20.03)		6.86 (2.50–18.81)		not sig		9.17 (3.37–24.96)	
	30.0–59.9	41.73 (25.17–69.19)		46.10 (25.75–82.51)		48.22 (23.65–98.31)		26.10 (9.31–73.17)		not sig		29.77 (10.69–82.88)	
	< 30	62.85 (21.00–188.06)		63.22 (17.83–224.14)		32.70 (4.09–261.59)		131.97 (24.14–721.62)		not sig		65.94 (7.36–590.56)	
Model 2^d^	> 90	Ref		Ref		Ref		Ref		Ref		Ref	
	69.0–89.9	1.38 (.82–2.34)		1.24 (.68–2.26)		1.09 (.52–2.29)		1.08 (.37–3.16)		not sig		2.03 (.71–5.80)	
	30.0–59.9	1.24 (.71–2.16)		1.09 (.58–2.07)		.98 (.45–2.15)		.97 (.30–3.13)		not sig		1.87 (.60–5.82)	
	< 30	1.68 (.55–5.17)		1.37 (.38–5.02)		.62 (.08–5.10)		4.16 (.69–24.99)		not sig		3.50 (.37–33.22)	
Model 3^e^	> 90	Ref		Ref		Ref		Ref		Ref		Ref	
	69.0–89.9	1.35 (.80–2.28)		1.20 (.65–2.20)		1.08 (.51–2.28)		.97 (.33–2.83)		not sig		2.00 (.70–5.74)	
	30.0–59.9	1.20 (.68–2.10)		1.05 (.55–2.00)		.98 (.44–2.16)		.81 (.25–2.63)		not sig		1.82 (.58–5.71)	
	< 30	1.64 (.53–5.05)		1.34 (.37–4.92)		.64 (.08–5.33)		3.10 (.51–18.79)		not sig		3.43 (.36–32.72)	
Model 4^f^	> 90	Ref		Ref		Ref		Ref		Ref		Ref	
	69.0–89.9	1.34 (.79–2.26)		1.18 (.64–2.16)		1.06 (.50–2.22)		.97 (.33–2.83)		not sig		2.04 (.71–5.82)	
	30.0–59.9	1.17 (.67–2.06)		1.02 (.54–1.94)		.96 (.43–2.12)		.75 (.23–2.44)		not sig		1.84 (.59–5.81)	
	< 30	1.57 (.51–4.85)		1.24 (.34–4.58)		.65 (.08–5.35)		2.29 (.37–14.08)		not sig		3.69 (.38–35.55)	
eGFR*Age													
	> 90	*Ref*		*Ref*		*Ref*		*Ref*		*Ref*		*Ref*	
	69.0–89.9	.95 (.91–.99)		*.97 (.92–1.02)*		*.96 (.90–1.02)*		.99 (.90–1.10)		*not sig*		.90 (.82–.99)	
	30.0–59.9	.90 (.86–.94)		.91 (.86–.96)		.90 (.84–.96)		.96 (.6–1.06)		*not sig*		.85 (.77–.94)	
	< 30	.88 (.79–.98)		.88 (.78–.98)		*.88 (.72–1.07)*		.91 (.77–1.06)		*not sig*		*.92 (.69–1.22)*	
eGFR*Sex													
	> 90	*Ref*		*Ref*		*Ref*		*Ref*		*Ref*		*Ref*	
	69.0–89.9	*1.98 (.72–5.44)*		3.25 (1.01–10.49)		*2.15 (.52–8.85)*		*9.98 (.98–101.33)*		*not sig*		*not sig*	
	30.0–59.9	*1.67 (.59–4.69)*		*2.80 (.85–9.30)*		*1.34 (.31–5.80)*		17.33 (1.63–183.87)		*not sig*		*not sig*	
	< 30	*2.58 (.29–23.32)*		*9.41 (.65–135.41)*		*.00 (.00–.00)*		*.00 (.00–.00)*		*not sig*		*not sig*	

^A^ Number of dementia cases

^B^ Age when examined in HUNT 2

^C^ Model 1: Categorized estimated Glomerular filtration rate (eGFR)

^D^ Model 2: eGFR, age, sex, education

^E^ Model 3: eGFR, age, sex, education, glomerular filtration rate, cholesterol, non-fasting blood glucose, serum iron, body mass index, pulse

^F^ Model 4: eGFR, age, sex, education, glomerular filtration rate, cholesterol, non-fasting blood glucose, serum iron, body mass index, pulse, history of myocardial infarction, diabetes mellitus, angina, stroke, smoking, subjective health status

### MA substudy

Study participants with a history of DM or using HTN medication, along with participants that did not have a history of DM or treated with HTN medication were examined both combined and in separate analyses. Characteristics are shown in Table 3 for the combined sample, and according to subgroups: DM or HTN and No DM or HTN, see Additional files 1 and 2. Mean ACR did not differ across quartiles of the total sample, and persons diagnosed with dementia were older, had higher SBP, DBP, lower renal function, and higher cholesterol. Table 4 shows the results of age and multivariate-adjusted analyses at different ACR levels expressed in quartiles. We found a positive association between increasing ACR and Combined AD and VaD. ACR in the fourth quartile (> 1.78 mg/mmol) with increased HR of VaD, 3.97 (1.12 to 14.07), compared with ACR in the first quartile (<.53 mg/mmol). In crude analyses, there were no sex interactions or age interactions between ACR and total dementia, combined AD/VaD, Mixed AD/VaD, and VaD.

**Table 3:** Characteristics of the moderately increased albuminuria sub study sample, examining albumin creatinine ratio (ACR)

HUNT 2 (1995–1997)	No dementia	Total Dementia	Combined AD/VaD/Mixed AD/VaD	AD	VaD	Mixed AD/VaD	Other Dementia	*P value* ^*A*^
Total Sample	6810	214	176	109	44	23	38	
ACR, mg/mmol mean (SD)	1.70 (3.06)	1.94 (3.37)	1.95 (3.47)	2.27 (4.31)	1.68 (1.20)	.97 (.50)	1.87 (2.89)	*.26*
ACR, quartiles, n (%)								*.01*
0–.53	1561 (22.9)	32 (15.0)	25 (14.2)	18 (16.5)	4 (9.1)	3 (13.0)	7(18.4)	
.54–.87	2232 (32.8)	70 (32.7)	58 (33.0)	36 (33.0)	10 (22.7)	12 (52.2)	12 (31.6)	
.88–1.77	1695 (24.9)	55 (25.7)	47 (26.7)	26 (23.9)	14 (31.8)	7 (30.4)	8 (21.1)	
1.78+	1322 (19.4)	57 (26.6)	46 (26.1)	29 (26.6)	16 (36.4)	1 (4.3)	11 (28.9)	
Diabetes Mellitus, n (%)	1075 (15.8)	31 (14.5)	28 (15.9)	16 (14.7)	7 (15.9)	5 (21.7)	3 (7.9)	*.61*
Antihypertensive tablets, n (%)	4569 (67.1)	168 (78.5)	136 (77.3)	80 (73.4)	40 (90.9)	16 (69.6)	32 (84.2)	*.00*
Sex, Female, n (%)	3665 (53.8)	133 (62.1)	114 (64.8)	77 (70.6)	22 (50.0)	15 (65.2)	19 (50.0)	*.02*
Age at HUNT 2 (1995–1997), mean (SD)	60.44 (14.74)	71.16 (6.49)	71.67 (6.43)	72.06 (6.16)	70.40 (7.25)	72.27 (5.91)	68.80 (6.37)	*.00*
Time to debut, years, mean (SD)		7.16 (3.74)	7.19 (3.85)	7.25 (4.03)	7.30 (3.82)	6.69 (3.06)	7.00 (3.22)	
Education, n (%)								*.01*
Primary	3766 (55.3)	141 (65.9)	120 (68.2)	76 (69.7)	28 (63.6)	16 (69.6)	21 (55.3)	
Completed secondary	2726 (40.0)	68 (31.8)	51 (29.0)	32 (29.4)	12 (27.3)	7 (30.4)	17 (44.7)	
Completed upper secondary	318 (4.7)	5 (2.3)	5 (2.8)	1 (.9)	4 (9.1)	0	0	
eGFR, mean (SD)	69.89 (15.63)	61.31 (12.76)	60.52 (12.53)	59.92 (11.76)	62.53 (15.31)	59.55 (10.05)	64.96 (13.34)	*.00*
Cholesterol (mmol/L), mean (SD)	6.24 (1.25)	6.69 (1.28)	6.78 (1.24)	6.87 (1.25)	6.70 (1.19)	6.48 (1.25)	6.27 (1.42)	*.00*
Non-fasting blood glucose (mmol/L), mean (SD)	6.19 (2.44)	6.30 (2.34)	6.28 (2.26)	6.36 (2.45)	6.19 (2.00)	6.05 (1.79)	6.42 (2.73)	*.51*
Serum iron (), mean (SD)	16.36 (5.98)	15.62 (5.50)	15.32 (5.26)	15.47 (5.19)	15.34 (5.57)	14.57 (5.17)	17.00 (6.38)	*.07*
BMI (kg/m^2^) mean (SD)	28.03 (4.54)	28.29 (4.31)	28.43 (4.20)	28.31 (4.04)	29.11 (4.92)	27.74 (3.44)	27.61 (4.78)	*.41*
Pulse (beats/min), mean (SD)	72.40 (13.64)	72.38 (13.64)	72.23 (12.77)	72.06 (12.88)	71.99 (1.78)	73.52 (14.47)	73.05 (17.31)	*.98*
Systolic BP (mmHg), mean (SD)	149.65 (23.29)	159.80 (24.26)	160.01 (21.19)	159.48 (23.49)	160.09 (24.45)	162.35 (27.74)	158.84 (24.90)	*.00*
Diastolic BP (mmHg), mean (SD)	85.27 (12.58)	88.34 (13.13)	88.22 (12.53)	87.24 (12.59)	90.36 (12.23)	88.74 (12.76)	88.89 (15.81)	*.00*
Myocardial Infarction, n (%)	581 (8.5)	19 (8.9)	13 (7.4)	10 (9.2)	3 (6.8)	0	6 (15.8)	*.86*
Angina Pectoris, n (%)	918 (13.5)	35 (16.4)	27 (15.3)	20 (18.3)	4 (9.1)	3 (13.0)	8 (21.1)	*.23*
Stroke, n (%)	332 (4.9)	16 (7.5)	14 (8.0)	5 (4.6)	8 (18.2)	1 (4.3)	2 (5.3)	*.08*
Daily Smoker, n (%)	1347 (19.8)	30 (14.0)	24 (13.6)	13 (11.9)	8 (18.2)	3 (13.0)	6 (15.8)	*.04*
Subjective health status								*.01*
Poor, n (%)	190 (2.8)	5 (2.3)	5 (2.8)	3 (2.8)	2 (4.5)	0	0	
Not so good, n (%)	2739 (40.2)	106 (49.5)	84 (47.7)	49 (45.0)	22 (50.0)	13 (56.5)	22 (57.9)	
Good, n (%)	3458 (50.8)	98 (45.8)	84 (47.7)	55 (50.5)	19 (43.2)	10 (43.5)	14 (36.8)	
Very good, n (%)	423 (6.2)	5 (2.3)	3 (1.7)	2 (1.8)	1 (2.3)	0	2 (5.3)	

^A^*P*-values are derived from *t* tests for continuous variables and *x*^2^ tests for the binary variables that compare means between total dementia and no dementia

**Table 4:** Cox proportional regression models on the association of albumin creatinine ratio (ACR) presented as quartiles and number of cases with dementia with its subgroups. Hazard ratios are presented with 95% confidence intervals

		Total Dementia	NC^A^	Combined AD, VaD, Mixed AD/VaD	NC	AD	NC	VaD	NC	Mixed AD/VaD	NC	Other Dementia	NC
DM or HTN medication^B^	ACR		184		151		90		42		19		33
Model 1^C^	0–.53	Ref		Ref		Ref		Ref		Ref		Ref	
	.54–.87	1.43 (.90–2.28)		1.64 (.96–2.80)		1.31 (.67–2.56)		2.03 (.55–7.51)		3.41 (.75–15.58)		.88 (.33–2.36)	
	.88–1.77	1.57 (.98–2.53)		**1.84 (1.07–3.16)**		1.31 (.67–2.56)		**3.89 (1.12–13.53)**		2.51 (.51–12.46)		.84 (.30–2.41)	
	1.78+	**2.29 (1.43–3.65)**		**2.55 (1.49–4.38)**		**2.14 (1.12–4.09)**		**5.92 (1.72–20.33)**		.55 (.05–6.12)		1.59 (.61–4.19)	
Model 2^D^	0–.53	Ref		Ref		Ref		Ref		Ref		Ref	
	.54–.87	1.36 (.85–2.17)		1.56 (.91–2.66)		1.25 (.66–2.39)		1.93 (.52–7.13)		3.19 (.70–14.57)		.84 (.31–2.26)	
	.88–1.77	1.24 (.77–2.01)		1.44 (.83–2.48)		1.01 (.52–1.98)		3.24 (.93–11.34)		1.79 (.36–8.96)		.70 (.24–2.01)	
	1.78+	**1.71 (1.06–2.75)**		**1.89 (1.10–3.26)**		1.60 (.83–1.98)		**4.52 (1.30–15.72)**		.37 (.36–8.96)		1.24 (.46–3.31)	
Model 3^E^	0–.53	Ref		Ref		Ref		Ref		Ref		Ref	
	.54–.87	1.38 (.86–2.20)		1.59 (.93–2.73)		1.28 (.67–2.46)		1.94 (.52–7.18)		3.29 (.72–15.16)		.82 (.30–2.21)	
	.88–1.77	1.22 (.75–1.98)		1.44 (.83–2.49)		1.04 (.52–2.04)		3.13 (.89–11.04)		1.69 (.33–8.54)		.65 (.22–1.88)	
	1.78+	**1.66 (1.03–2.69)**		**1.87 (1.08–3.25)**		1.61 (.83–3.13)		**4.35 (1.24–15.28)**		.34 (.03–3.86)		1.12 (.41–3.04)	
Model 4^F^	0–.53	Ref		Ref		Ref		Ref		Ref		Ref	
	.54–.87	1.38 (.86–2.21)		1.60 (.93–2.74)		1.30 (.68–2.50)		1.82 (.49–6.76)		3.78 (.82–17.54)		.83 (.31–2.24)	
	.88–1.77	1.23 (.76–2.00)		1.46 (.84–2.53)		1.07 (.54–2.12)		2.93 (.83–10.40)		1.87 (.37–9.53)		.61 (.21–1.78)	
	1.78+	**1.65 (1.02–2.69)**		**1.85 (1.06–3.23)**		1.66 (.85–3.24)		**3.97 (1.12–14.07)**		.36 (.03–4.08)		1.12 (.41–3.09)	
ACR*Age		*.11*		*.09*		*.91*		*.15*		*.24*		*.86*	
ACR*Sex		*.46*		*.38*		*.44*		*.77*		*.20*		*.09*	

^A^ Number of dementia cases

^B^ Self-reported history of Diabetes mellitus (DM) or hypertension (HTN) medication users

^C^ Model 1: albumin creatinine ratio, quartiles (ACR)

^D^ Model 2: ACR, age, sex, education

^E^ Model 3: ACR, age, sex, education, glomerular filtration rate, cholesterol, non-fasting blood glucose, serum iron, body mass index, pulse

^F^ Model 4: ACR, age, sex, education, glomerular filtration rate, cholesterol, non-fasting blood glucose, serum iron, body mass index, pulse, history of myocardial infarction, diabetes mellitus, angina, stroke, smoking, subjective health status

Bold entries where adverse and inverse associations which are significant in this table

## Discussion

The main finding of this study was that VaD and AD was positively associated with MA, expressed as ACR, in participants under 72 years of age, amongst a treated hypertensive or diagnosed with DM sample. However, despite having a study period of up to 16 years in this large population sample, we found no increased risk of dementia with decreasing eGFR regardless of DM, HTN or no illness status. Our findings contribute to a number of studies examining the association between dementia and renal disease, using ACR and eGFR as markers [11, 20–23]. Our Norwegian study contrasts with one of the first prospective studies that was performed by Miwa et al. examining 660 Japanese subjects that found an association between eGFR and incident dementia [24]. This contrasting finding could be attributed to different study populations as our study subjects had lower vascular risk compared with the Japanese subjects. Our study supports findings from the 3C Study, where eGFR values were not associated with an increased risk of incident dementia or cognitive decline over the seven-year follow-up^11^. Although our study featured a long follow-up, we also had a relatively low percentage of participants with eGFR < 60 ml/min). Another prospective study by Tamura et al. also found no significant association between cognitive impairment and risk for progression of CKD [25].

MA is a known pre-cursor for progressive renal damage, diabetic nephropathy, and has been reported to occur in approximately 15–30% of hypertensive patients [15, 26, 27]. There are few studies that have examined the association between MA and dementia, as most studies have data reporting on severely increased albuminuria, defined with ACR > 30 mg/mmol. A recent comparative prospective community-based cohort study has revealed a significant association between albuminuria and risk for development of AD and VaD. Furthermore, a recent systematic review and meta-analyses has concluded that albuminuria was independently associated with cognitive impairment, dementia and cognitive decline [8]. Georgakis et al. showed stronger effects for vascular dementia and cognitive performance in areas primarily affected by microvascular disease and concluded to support the association could be mediated by shared microvascular pathology in the kidney and the brain [28].

Although pathophysiological mechanisms are largely unknown, there are many hypotheses describing possible mechanisms [29, 30]. Nitric oxide (NO) deficiency regulates the microcirculation and blood brain barrier. Patients with impaired cognition show elevated levels of endogenous inhibitors of NO synthesis and decreased NO metabolites, presumably a result of CKD [31]. Strain vessel hypothesis is another presumed mechanism that vessels are exposed to very high pressure, and maintain a high vascular tone. These strained vessels are recognized to share similar pressure-induced injuries, where MA is speculated to be a marker of cerebrovascular-renal injury [3, 32]. These two end organs share similar anatomic and hemodynamic features and vessels are shown to undergo similar mechanisms responding to vascular strain [33]. Juxtamedullary afferent arterioles in the kidney and perforating arterioles in the brain are downstream from high-pressure arteries, and both organs are vulnerable to hypertensive vascular damage [34]. Cerebro-renal interactions are presumed to exist as clinical studies have found a higher prevalence of vascular cognitive impairment in participants with reduced kidney function [24, 34, 35]. Microvascular damage has shown to alter the hemodynamics of the neurovasculature that contributes to cognitive changes seen in the early stages of dementia [35, 36]. Small vessel disease in the kidney may therefore also indicate the presence of small vessel disease in the brain [24, 33, 37].

This study adjusted the effects of MA for eGFR, which is seldom in previously published studies. Participants diagnosed with dementia were an average of 77 years of age when diagnosed with dementia. Despite this relatively young age, MA is still a risk factor for developing dementia. The majority of participants were treated HTN subjects and/or with DM along with a random sample without DM and were not treated with HTN medication. The population-based approach and high attendance rate make selection bias less likely. MA analyses were performed with fresh urine samples without long-term storage, and used data from an average of three urine samples.

This study has limitations. Competing risk from death and other causes is an unavoidable limitation in studies examining participants with older age. Furthermore, older aged participants with lower eGFR are at higher risk of mortality than participants with higher eGFR are, and may therefore never develop incident dementia. Results show that the hazards are identical between groups for dementia, and both the CHR and SHR models estimate the effects as null, as expected. This study did not have access to the national prescription register that provides specific details on types of medication taken by participants. Therefore, data used in analyses was based on self-reported history. As mentioned, the number of participants with CKD was low, as only 12.2% had eGFR < 60 mL. In addition, ACR was measured only in a subsample of the HUNT 2 sample. Experienced clinicians used standardized ICD-10 criteria to ascertain the subtypes of dementia diagnoses, but this was retrospective and based on comprehensive medical records from both hospital and nursing home panels. Data inspection revealed that the period during study startup in 1995–2000 showed infrequent numbers of dementia cases. A greater number of dementia cases were diagnosed during 2000–2009, and the greatest amount of cases with dementia were identified in 2009–2011. Additionally, cognition assessment was not a standard evaluation during HUNT 2 at baseline, and one must note that there was no access to data from individuals with dementia who were under the care of their general practitioner, and these will appear as false-negatives in the data set. It is uncertain if this would influence the results substantially, but unlikely as the proportion of false negative is quite low because the prevalence of dementia is low.

## Conclusion

Our results strengthens the hypothesis that vascular mechanisms may affect both kidney and brain as an association between MA, VaD, and Combined AD/VaD was found. However, eGFR was not significantly associated with dementia independent of DM or HTN.

## Additional files

Additional file 1: Characteristics of subsample study examining albumin creatinine ratio amongst participants who self-reported history of diabetes mellitus or taking anti-hypertensive medication. (DOCX 21 kb)

Additional file 2: Characteristics of subsample study examining albumin creatinine ratio amongst participants who self-reported no history of diabetes mellitus or did not report taking anti-hypertensive medication. (DOCX 30 kb)

Additional file 3: Estimated cause specific hazard (CHR) and subdistribution hazard ratios (SHR) for death and dementia using multivariate regression model. Cox regression cause specific hazards (CHR) and Fine-Gray subdistribution hazards (SHR) are shown with 95% CI. (DOCX 28 kb)

## Data Availability

The funding institutions are currently granted the exclusive privilege to perform studies on data derived from the HMS Study. Future collaboration with researchers outside the current research group will be welcomed later. Information about application procedures is available at hunt@medisin.ntnu.no.

## Abbreviations

ACR: Albumin creatinine ratio
AD: Alzheimer disease;
BMI: Body mass index
BP: Blood pressure
CHR: Cause specific hazard ratio
CI: Confidence interval
CKD: Chronic kidney disease
CKD-EPI: Chronic Kidney Disease Epidemiology Collaboration
DBP: Diastolic blood pressure
DM: Diabetes mellitus
eGFR: Estimated glomerular filtration rate
FTD: Frontal temporal dementia
HMS: Health and Memory Study of Nord-Trøndelag County
HR: Hazard ratio
HTN: Hypertension
HUNT: The Health Study of Nord-Trøndelag
ICD-10: International classification of diseases
LBD: Lewy body dementia
MA: Moderately increased albuminuria
MCI: Mild cognitive impairment
MI: Myocardial infarction
Mixed AD/VaD: Mixed Alzheimer’s Disease/vascular dementia
NO: Nitric oxide
NTNU: Norwegian University of Science and Technology
REK: Regional Committee for Medical and Health Research Ethics
SBP: Systolic blood pressure
SH: Subdistribution hazard
SHR: Subdistribution hazard ratio
SPSS: Statistical Package for the Social Sciences
VaD: Vascular dementia
