# Supplementary material for: Moderately increased albuminuria, chronic kidney disease and incident dementia: the HUNT study
Source: BMC Nephrol. 2019 Jul 12;20:261. doi: 10.1186/s12882-019-1425-8 (PMC6626412; doi:10.1186/s12882-019-1425-8)
Supplement: Supplementary file 1 — Characteristics of subsample study examining albumin creatinine ratio amongst participants who self-reported history of diabetes mellitus or taking anti-hypertensive medication. (DOCX 21 kb) [file 12882_2019_1425_MOESM1_ESM.docx]

Additional file 1. Characteristics of Microalbuminuria study (MA) examining albumin creatinine ratio (ACR) amongst participants who self-reported history of diabetes mellitus or taking anti-hypertensive medication.

| **HUNT 2 (1995-1997) MA Substudy** | No dementia | Total Dementia | Combined AD/VaD/Mixed AD/VaD | AD | VaD | Mixed AD/VaD | Other Dementia | ***P value*** |
| --- | --- | --- | --- | --- | --- | --- | --- | --- |
|  |  |  |  |  |  |  |  |  |
|  | 5 135 | 184 | 151 | 90 | 42 | 19 | 33 |  |
| ACR, mean (SD) | 1.92 (3.36) | 2.10 (3.60) | 2.12 (3.72) | 2.54 (4.69) | 1.73 (1.21) | 1.00 (.53) | 1.98 (3.08) | .49 |
| ACR, quartiles, n (%) |  |  |  |  |  |  |  | *.11* |
| 0-.53 | 1 034 (20.1) | 26 (14.1) | 19 (12.6) | 14 (15.6) | 3 (7.1) | 2 (10.5) | 7 (21.2) |  |
| .54-.87 | 1 539 (30.0) | 55 (29.9) | 46 (30.5) | 27 (30.0) | 9 (21.4) | 10 (52.6) | 9 (27.3) |  |
| .88-1.77 | 1 366 (26.6) | 49 (26.6) | 42 (27.8) | 22 (24.4) | 14 (33.3) | 6 (31.6) | 7 (21.2) |  |
| 1.78+ | 1 196 (23.3) | 54 (29.3) | 44 (29.1) | 27 (30.0) | 16 (38.1) | 1 (5.3) | 10 (30.3) |  |
| Diabetes Mellitus, n (%) | 1 075 (20.9) | 31 (16.8) | 28 (18.5) | 16 (17.8) | 7 (16.7) | 5 (26.3) | 3 (9.1) | .18 |
| Antihypertensive tablets, n (%) | 4 569 (89.0) | 168 (91.3) | 136 (90.1) | 80 (88.9) | 40 (95.2) | 16 (84.2) | 32 (97.0) | *.32* |
| Sex, Female, n (%) | 2 782 (54.2) | 114 (62.0) | 97 (64.2) | 62 (68.9) | 22 (52.4) | 13 (68.4) | 17 (51.5) | *.04* |
| Age at HUNT 2 (1995-1997), mean (SD) | 64.54 (12.13) | 71.12 (6.37) | 71.63 (6.23) | 72.03 (5.78) | 70.11 (7.25) | 73.08 (5.46) | 68.78 (6.58) | *.00* |
| Time to debut, years, mean (SD) |  | 7.17 (3.80) | 7.19 (3.90) | 7.21 (4.09) | 7.24 (7.25) | 6.97 (3.16) | 7.07 (6.58) |  |
| Education, n (%) |  |  |  |  |  |  |  | .38 |
| Primary | 3 201 (62.3) | 122 (66.3) | 103 (68.2) | 62 (68.9) | 27 (64.3) | 14 (73.7) | 19 (57.6) |  |
| Completed secondary | 1 743 (33.9) | 58 (31.5) | 44 (29.1) | 27 (30.0) | 12 (28.6) | 5 (26.3) | 14 (42.4) |  |
| Completed upper secondary | 191 (3.7) | 4 (2.2) | 4 (2.6) | 1 (1.1) | 3 (7.1) | 0 | 0 |  |
| estimated glomerular filtration rate (eGFR), mean (SD) | 66.38 (14.38) | 60.85 (13.35) | 59.99 (13.11) | 59.16 (12.37) | 62.34 (15.45) | 58.73 (10.69) | 64.77 (13.93) | *.00* |
| Cholesterol (mmol/L), mean (SD) | 6.37 (1.24) | 6.67 (1.34) | 6.76 (1.30) | 6.85 (2.65) | 6.70 (1.20) | 6.47 (1.36) | 6.28 (1.47) | *.00* |
| Non-fasting blood glucose (mmol/L), mean (SD) | 6.47 (2.66) | 6.42 (2.49) | 6.38 (2.39) | 6.48 (2.65) | 6.25 (2.03) | 6.21 (1.88) | 6.59 (2.90) | *.77* |
| Serum iron (mmol/L), mean (SD) | 16.33 (5.95) | 15.84 (5.66) | 15.50 (5.41) | 15.73 (5.30) | 15.29 (5.70) | 14.84 (5.46) | 17.42 (6.54) | *.28* |
| Body Mass Index (kg/m^2^) mean (SD) | 28.69 (4.59) | 28.65 (4.26) | 28.81 (4.09) | 28.83 (3.97) | 29.30 (4.88) | 27.66 (15.12) | 27.89 (4.95) | *.89* |
| Pulse (beats/min), mean (SD) | 72.23 (14.07) | 71.76 (13.89) | 71.41 (13.01) | 71.06 (13.14) | 71.68 (11.96) | 72.53 (15.12) | 73.32 (17.54) | *.65* |
| Systolic BP (mmHg), mean (SD) | 154.58 (22.24) | 160.89 (23.81) | 161.09 (23.86) | 160.17 (23.20) | 161.21 (24.47) | 165.16 (26.40) | 160.00 (23.97) | *.00* |
| Diastolic BP (mmHg), mean (SD) | 87.19 (12.38) | 88.61 (12.75) | 88.46 (12.16) | 87.18 (11.94) | 91.21 (11.84) | 88.47 (13.51) | 89.30 (15.34) | *.13* |
| Myocardial Infarction, n (%) | 549 (10.7) | 18 (9.8) | 12 (7.9) | 9 (10.0) | 3 (7.1) | 0 | 6 (18.2) | *.70* |
| Angina Pectoris, n (%) | 881 (17.2) | 33 (17.9) | 26 (17.2) | 19 (21.1) | 4 (9.5) | 3 (15.8) | 7 (21.2) | *.78* |
| Stroke, n (%) | 322 (6.3) | 16 (8.7) | 14 (9.3) | 5 (5.6) | 8 (19.0) | 1 (5.3) | 2 (6.1) | *.19* |
| Daily Smoker, n (%) | 864 (16.8) | 25 (13.6) | 19 (12.6) | 11 (12.2) | 7 (16.7) | 1 (5.3) | 6 (18.2) | *.25* |
| Subjective health status |  |  |  |  |  |  |  | *.43* |
| Poor, n (%) | 174 (3.4) | 4 (2.2) | 4 (2.6) | 2 (2.2) | 2 (4.8) | 0 | 0 |  |
| Not so good, n (%) | 2 391 (46.6) | 95 (51.6) | 75 (49.7) | 43 (47.8) | 21 (50.0) | 11 (57.9) | 20 (60.6) |  |
| Good, n (%) | 2 433 (47.4) | 82 (44.6) | 69 (45.7) | 43 (47.8) | 18 (42.9) | 8 (42.1) | 13 (39.4) |  |
| Very good, n (%) | 137 (2.7) | 3 (1.6) | 3 (2.0) | 2 (2.2) | 1 (2.4) | 0 | 0 |  |
| ^A^*P*-values are derived from *t* tests for continuous variables and *x*^2^ tests for the binary variables between columns: total dementia and no dementia. | | | | | | | | |
