# Supplementary material for: Moderately increased albuminuria, chronic kidney disease and incident dementia: the HUNT study
Source: BMC Nephrol. 2019 Jul 12;20:261. doi: 10.1186/s12882-019-1425-8 (PMC6626412; doi:10.1186/s12882-019-1425-8)
Supplement: Supplementary file 2 — Characteristics of subsample study examining albumin creatinine ratio amongst participants who self-reported no history of diabetes mellitus or did not report taking anti-hypertensive medication. (DOCX 30 kb) [file 12882_2019_1425_MOESM2_ESM.docx]

Additional file 2: Characteristics of Microalbuminuria (MA) study examining albumin creatinine ratio (ACR) amongst participants who self-reported no history of diabetes mellitus or did not report taking anti-hypertensive medication.

| **HUNT 2 (1995-1997)**  **MA Substudy** | No dementia | Total Dementia | Combined AD/VaD/Mixed AD/VaD | AD | VaD | Mixed AD/VaD | Other Dementia | ***P value*** |
| --- | --- | --- | --- | --- | --- | --- | --- | --- |
|  |  |  |  |  |  |  |  |  |
| No DM or BP medication Sample | 1 675 | 30 | 25 | 19 | 2 | 4 | 5 |  |
| ACR, mean (SD) | 1.00 (1.69) | .97 (.70) | .93 (.69) | .99 (.77) | .58 (.12) | .85 (.42) | 1.15 (.82) | *.93* |
| ACR, quartiles, n (%) |  |  |  |  |  |  |  | *.57* |
| 0-.53 | 527 (31.5) | 6 (20.0) | 6 (24.0) | 4 (21.1) | 1 (50.0) | 1 (25.0) | 3 (60.0) |  |
| .54-.87 | 693 (41.4) | 15 (50.0) | 12 (48.0) | 9 (47.4) | 1 (50.0) | 2 (50.0) | 1 (20.0) |  |
| .88-1.77 | 329 (19.6) | 6 (20.0) | 5 (20.0) | 4 (21.1) | 0 | 1 (25.0) | 0 |  |
| 1.78+ | 126 (7.5) | 3 (10.0) | 2 (8.0) | 2 (10.5) | 0 | 0 | 1 (20.0) |  |
| Diabetes Mellitus, n (%) | 0 | 0 | 0 | 0 | 0 | 0 | 0 |  |
| Antihypertensive tablets, n (%) | 0 | 0 | 0 | 0 | 0 | 0 | 0 |  |
| Sex, Female, n (%) | 883 (52.7) | 19 (63.3) | 17 (68.0) | 15 (78.9) | 0 | 2 (50) | 2 (40) | *.25* |
| Age at HUNT 2 (1995-1997), mean (SD) | 47.86 (14.93) | 71.43 (7.32) | 71.93 (7.64) | 72.19 (7.9) | 76.50 (4.95) | 68.43 (7.29) | 68.92 (5.39) | *.00* |
| Time to debut, years, mean (SD) |  | 7.08 (3.37) | 7.20 (3.57) | 7.43 (3.84) | 8.60 (2.26) | 5.38 (2.45) | 6.52 (2.31) |  |
| Education, n (%) |  |  |  |  |  |  |  | .00 |
| Primary | 565 (33.7) | 19 (63.3) | 17 (68.0) | 14 (73.7) | 1 (50) | 2 (50) | 2 (40) |  |
| Completed secondary | 983 (58.7) | 10 (33.3) | 7 (28.0) | 5 (26.3) | 0 | 2 (50) | 3 (60) |  |
| Completed upper secondary | 127 (7.6) | 1 (3.3) | 1 (4.0) | 0 | 1 (50) | 0 | 0 |  |
| estimated glomerular filtration rate (eGFR), mean (SD) | 80.64 (14.46) | 64.16 (7.85) | 63.74 (7.64) | 63.52 (7.55) | 66.44 (16.27) | 63.45 (5.53) | 66.25 (9.52) | *.00* |
| Cholesterol (mmol/L), mean (SD) | 5.84 (1.19) | 6.78 (.87) | 6.90 (.78) | 7.00 (.77) | 6.70 (1.41) | 6.53 (.56) | 6.20 (1.17) | *.00* |
| Non-fasting blood glucose (mmol/L), mean (SD) | 5.31 (1.22) | 5.60 (.89) | 5.65 (.95) | 5.78 (.94) | 5.10 (.85) | 5.30 (1.09) | 5.32 (.42) | *.21* |
| Serum iron (mmol/L), mean (SD) | 16.46 (6.08) | 14.23 (4.21) | 14.24 (4.21) | 14.21 (4.52) | 16.50 (.71) | 13.3 (3.78) | 14.20 (4.71) | *.04* |
| Body Mass Index (kg/m^2^) mean (SD) | 25.99 (3.69) | 26.08 (4.03) | 26.14 (4.24) | 25.84 (3.45) | 25.15 (5.73) | 28.10 (7.46) | 25.76 (3.10) | *.89* |
| Pulse (beats/min), mean (SD) | 72.92 (12.22) | 76.20 (11.45) | 77.18 (10.07) | 76.82 (10.61) | 78.50 (4.24) | 78.25 (11.24) | 71.30 (17.50) | *.14* |
| Systolic BP (mmHg), mean (SD) | 134.56 (19.67) | 153.10 (26.26) | 153.48 (25.63) | 156.21 (25.21) | 136.50 (2.12) | 149.00 (34.26) | 151.20 (32.44) | *.00* |
| Diastolic BP (mmHg), mean (SD) | 79.36 (11.26) | 86.63 (15.42) | 86.72 (14.75) | 87.53 (15.71) | 72.50 (4.95) | 90.00 (9.87) | 86.20 (20.46) | *.02* |
| Myocardial Infarction, n (%) | 32 (1.9) | 1 (3.3) | 1 (4.0) | 1 (5.3) | 0 | 0 | 0 | *.58* |
| Angina Pectoris, n (%) | 37 (2.2) | 2 (6.7) | 1 (4.0) | 1 (5.3) | 0 | 0 | 1 (20.0) | *.11* |
| Stroke, n (%) | 10 (.6) | 0 | 0 | 0 | 0 | 0 | 0 | *.67* |
| Daily Smoker, n (%) | 483 (28.8) | 5 (16.7) | 5 (20.0) | 2 (10.5) | 1 (50) | 2 (50.0) | 0 | *.14* |
| Subjective health status |  |  |  |  |  |  |  | *.06* |
| Poor, n (%) | 16 (1.0) | 1 (3.3) | 1 (4.0) | 1 (5.3) | 0 | 0 | 0 |  |
| Not so good, n (%) | 348 (20.8) | 11 (36.7) | 9 (36.0) | 6 (31.6) | 1 (50.0) | 2 (50.0) | 2 (40.0) |  |
| Good, n (%) | 1 025 (61.2) | 16 (53.3) | 15 (60.0) | 12 (63.2) | 1 (50.0) | 2 (50.0) | 1 (20) |  |
| Very good, n (%) | 286 (17.1) | 2 (6.7) | 0 | 0 | 0 | 0 | 2 (40) |  |
| ^A^*P*-values are derived from *t* tests for continuous variables and *x*^2^ tests for the binary variables between columns: total dementia and no dementia. | | | | | | | | |
