# Supplementary material for: Moderately increased albuminuria, chronic kidney disease and incident dementia: the HUNT study
Source: BMC Nephrol. 2019 Jul 12;20:261. doi: 10.1186/s12882-019-1425-8 (PMC6626412; doi:10.1186/s12882-019-1425-8)
Supplement: Supplementary file 3 — Estimated cause specific hazard (CHR) and subdistribution hazard ratios (SHR) for death and dementia using multivariate regression model. Cox regression cause specific hazards (CHR) and Fine-Gray subdistribution hazards (SHR) are shown with 95% CI. (DOCX 28 kb) [file 12882_2019_1425_MOESM3_ESM.docx]

Additional file 3: Comparison of Cox regression and Fine and Gray. Cause specific hazard ratios (CHR) were from the Cox regression. Subdistribution hazard ratios (SHR) were obtained using the Fine-Gray model.

| Competing Risk Regression^A^ | **Event =Death** | | | | **Event =Dementia** | | | |
| --- | --- | --- | --- | --- | --- | --- | --- | --- |
|  | Cox | | Fine and Gray | | Cox | | Fine and Gray | |
|  | CHR | 95% CI | SHR | 95% CI | CHR | 95% CI | SHR | 95% CI |
| Total Dementia, n=668 |  |  |  |  |  |  |  |  |
| eGFR >90 | Ref | Ref | Ref | Ref | Ref | Ref | Ref | Ref |
| 60-89 | **.68** | **(.61-.77)** | **.69** | **(.62-.77)** | 1.38 | (.82-2.33) | 1.76 | (1.03-2.99) |
| 30-59 | **.80** | **(.70-.91)** | **.79** | **(.70-.90)** | 1.24 | (.71-2.16 | 1.43 | (.80-2.55) |
| <30 | **1.60** | **(1.23-2.08)** | **1.52** | **(1.14-2.05)** | 1.68 | (.55-5.17) | 1.22 | (.39-3.85) |
| AD, n=348 |  |  |  |  |  |  |  |  |
| eGFR >90 | Ref | Ref | Ref | Ref | Ref | Ref | Ref | Ref |
| 60-89 | **.69** | **(.61-.77)** | **.69** | **(.61-.77)** | 1.09 | (.52-2.29) | 1.43 | (.68-3.02) |
| 30-59 | **.80** | **(.70-.91)** | **.80** | **(.71-.91)** | .98 | (.45-2.15) | 1.17 | (.52-2.63) |
| <30 | **1.63** | **(1.26-2.12)** | **1.67** | **(1.27-2.20)** | .62 | (.08-5.09) | .45 | (.05-3.78) |
| VaD, n=112 |  |  |  |  |  |  |  |  |
| eGFR >90 | Ref | Ref | Ref | Ref | Ref | Ref | Ref | Ref |
| 60-89 | **.69** | **(.61-.77)** | **.68** | **(.61-.77)** | 1.08 | (.37-3.16) | 1.38 | (.46-4.13) |
| 30-59 | **.80** | **(.70-.90)** | **.79** | **(.70-.90)** | .97 | (.30-3.13) | 1.10 | (.32-3.80) |
| <30 | **1.59** | **(1.22-2.07)** | **1.51** | **(1.12-2.04)** | 4.16 | (.69-24.99) | 2.96 | (.45-19.35) |
| Mixed AD/VaD, n=68 |  |  |  |  |  |  |  |  |
| eGFR >90 | Ref | Ref | Ref | Ref | Ref | Ref | Ref | Ref |
| 60-89 | **.69** | **(.61-.77)** | **.69** | **(.61-.77)** | not sig | not sig | not sig | not sig |
| 30-59 | **.80** | **(.70-.91)** | **.80** | **(.70-.90)** | not sig | not sig | not sig | not sig |
| <30 | **1.63** | **(1.26-2.12)** | **1.64** | **(1.24-2.17)** | not sig | not sig | not sig | not sig |
| Combined AD/VaD/Mixed AD/VaD, n=528 |  |  |  |  |  |  |  |  |
| eGFR >90 | Ref | Ref | Ref | Ref | Ref | Ref | Ref | Ref |
| 60-89 | **.68** | **(.61-.77)** | **.69** | **(.61-.77)** | 1.24 | (.68-2.26) | 1.60 | (.87-2.94) |
| 30-59 | **.80** | **(.70-.91)** | **.79** | **(.70-.90)** | 1.09 | (.58-2.07) | 1.28 | (.66-2.49) |
| <30 | **1.60** | **(1.24-2.08)** | **1.56** | **(1.17-2.10)** | 1.37 | (.38-5.02) | 1.01 | (.27-3.81) |
| Other Dementia, n=140 |  |  |  |  |  |  |  |  |
| eGFR >90 | Ref | Ref | Ref | Ref | Ref | Ref | Ref | Ref |
| 60-89 | **.69** | **(.61-.77)** | **.69** | **(.61-.77)** | 2.03 | (.71-5.80) | 2.47 | (.83-7.32) |
| 30-59 | **.80** | **(.70-.91)** | **.80** | **(.70-.90)** | 1.87 | (.60-5.82) | 2.02 | (.60-6.83) |
| <30 | **1.63** | **(1.25-2.11)** | **1.58** | **(1.19-2.10)** | 3.50 | (.37-33.21) | 2.38 | (.24-23.18) |

^A^eGFR, controlled for age, sex, and education
